# Is Cognitive Reserve a Determinant of Functional and Mental Health in Older People of the Sardinian Blue Zone? A Mediational Approach

**DOI:** 10.1007/s11126-023-10047-6

**Published:** 2023-08-29

**Authors:** Maria Chiara Fastame, Benedetta Brandas, Massimiliano Pau

**Affiliations:** 1 Department of Pedagogy, Psychology, Philosophy, University of Cagliari, Via Is Mirrionis 1, Cagliari, 09123 Italy; 2 Department of Mechanical, Chemical, and Materials Engineering, University of Cagliari, Via Marengo 2, Cagliari, 09123 Italy

**Keywords:** Depressive symptoms, Cognitive reserve, Motor efficiency, Elders, Blue Zone, Aging

## Abstract

The main purpose of this study was to examine the mediating role of cognitive reserve in the relationship between functional health (expressed through the amount and intensity of performed physical activity objectively assessed using wearable accelerometers) and psychological well-being (i.e., assessed in terms of self-reported depressive signs) of older people living in an area of exceptional longevity, the so-called Sardinian Blue Zone. A further goal was to investigate the impact of gender on the cognitive reserve and physical health of our participants, using global cognitive functioning as a covariate. A battery of tests assessing motor efficiency, cognitive reserve, global cognitive functioning, and self-reported depressive symptoms was individually presented to 120 community dwellers (M_age_ = 82 years, SD = 8.4 years) of the Sardinian Blue Zone. Significant associations were found between cognitive reserve, motor efficiency, and self-reported depressive signs. Moreover, three mediation analyses documented that distinct indexes of cognitive reserve and motor efficiency explain 27.2-31% of the variance in the self-reported depression condition. Following this, it was also found that people with scarce cognitive reserve tended to exhibit significant signs of depression and showed worse motor abilities. In addition, after controlling for the effect of global cognitive functioning, motor efficiency, and cognitive reserve were generally more preserved in males than in females. Overall, these findings suggest that cognitive reserve is a compensatory resource that contributes significantly to the enhancement of health-related quality of life in the last decades of life.

**Supplementary Information:**

The online version contains supplementary material available at 10.1007/s11126-023-10047-6.

## Introduction

According to the World Health Organization [1] nowadays individuals live approximately 20 years longer than 50 years ago. In 2030 older people (i.e., sexagenarians and older individuals) are expected to be 1.4 billion, and by 2050 they are estimated to be approximately 2.1 billion [2]. Thus, the rapid aging of the global population is a very challenging demographic phenomenon that requires the implementation of specific actions promoting health-related quality of life (HRQoL) in late adulthood.

Despite the lack of a consensual definition, HRQoL may be defined as the subjective dimension of quality of life, which encompasses a sub-set of feelings on an individual’s health that, in turn, impact his/her perceived psychological well-being, his/her functional abilities to perform some daily activities (e.g., motor efficiency) and to be engaged in social life [3, 4]. Accordingly, it is crucial to focus on the factors determining HRQoL, since it is an important dimension of successful aging, a dynamic process in which one’s overall subjective mental and functional health is central [5]. From an applied perspective, the enhancement of HRQoL in later life implies the early identification of the potential factors favoring (or menacing) successful aging by studying the functional and psychological phenotype of populations aging exceptionally well. In this regard, Poulain et al. [6] validated five isolated areas in the world characterized by the exceptional longevity of their inhabitants, the so-called Blue Zones (BZ), which are located in the central-eastern and mountainous region of Sardinia (Italy), in the Nicoya Peninsula (Costa Rica), Ikaria (Greece), Okinawa (Japan), and Noma Linda (USA).

A relevant role in ensuring successful aging is played by regular physical activity (PA), as has been reported in several large epidemiological studies which demonstrated the protective effects of an active lifestyle against major chronic diseases, cognitive impairments, limitations of physical functions associated with activities of daily living and mental health disorders [7–9]. Interestingly, such positive effects were observed not only in the case of structured PA but also (although with different magnitude) when older individuals were engaged in high-demand leisure activities such as walking, gardening, swimming, etc. [10]. In this regard, it is noteworthy to observe that even a simple walking activity can be very beneficial in terms of successful aging, as higher steps volume and intensity, objectively assessed using three-axial accelerometers, are significantly associated with lower hospitalization, reduced risk of all-cause mortality in community-dwelling older adults [11], better cognitive functioning [12], and lower risk of all-cause dementia [13]. Extending this evidence, it has been reported that longevous people living in Ikaria and the Sardinian BZ are regularly engaged in moderate PA (e.g., 12,000 steps per day) that significantly contributes to their successful aging and perceived functional health [14, 15].

As regards mental well-being in late adulthood, there is evidence that approximately 94% of the centenarians living in Nicoya have reported being satisfied with their life [16]. Similarly, older people of the Sardinian BZ have shown greater life satisfaction and lower depressive symptoms than their peers recruited in the Sardinian capital town of Cagliari [17] or those living in other rural areas located in Sardinia [18] and northern Italy [19]. Moreover, one outcome that older people of the Sardinian BZ share with those of Ikaria is that in both those contexts, depressive symptoms were significantly below the national cut-off values [e.g., 19–21], suggesting that mental well-being is a crucial aspect of HRQoL to age well. In addition, it has been shown that older women weavers living in Okinawa who were engaged in regular PA to contribute to the production of local handcrafts were more longevous, and reported very high levels of physical health and mental well-being [22]. Consistent with this, older and longevous people of the Sardinian BZ, Okinawa, and Ikaria who were regularly engaged in socio-cultural and physical activities (e.g., gardening, walking) have reported better perceived physical health, greater psychological well-being, more preserved cognitive functioning, and were more socially active within their communities than sedentary peers [18, 20, 23–25]. Overall, these findings are consistent with a trend of research showing that cognitive reserve is a protective factor for HRQoL in the late adult span (e.g., maintaining independence, avoiding depression) and therefore for successful aging [26].

Cognitive reserve refers to “the adaptability (i.e., efficiency, capacity, flexibility of cognitive processes) that helps to explain differential susceptibility of cognitive abilities or day-to-day function to brain aging, pathology, or insult” (p. 1306) [27]. Therefore, when aging or some neurodegenerative disorders impact brain functioning, a higher cognitive reserve is a compensatory resource that can be helpful to cope with brain deterioration in late adulthood [28]. Cognitive reserve is usually assessed indirectly in terms of single proxies (i.e., years of education, work occupation, and level of engagement in leisure activities) or through some psychological inventories developed to provide an index that summarizes the different dimensions of this construct. In this regard, there is evidence that a general measure of cognitive reserve assessed through the Cognitive Reserve Index questionnaire [29] and its specific dimension of leisure time are inversely associated with self-reported depressive symptoms in older adults [30]. Extending this, it has also been documented the significant association between cognitive reserve and physical reserve [31], that is, older people exhibiting better functional health assessed in terms of handgrip strengths and gait speed also reported a greater score in the Cognitive Reserve Index [32]. In addition, it has been shown that aerobic exercise promotes the maintenance of good functional health, cognitive efficiency, and psychological well-being (e.g., fewer depressive symptoms) in late adulthood [e.g., 33, 34]. Extending this, a recent longitudinal study conducted among older individuals of the Sardinian BZ documented that reduced self-reported depressive signs were associated with higher objectively assessed mobility parameters (e.g., gait speed) both at baseline and after 24 months [35].

Despite the body of research that has documented the impact of cognitive and functional reserves on HRQOL in the last decades of life, to our knowledge, no studies have concurrently examined the relationship between cognitive reserve, functional health, and psychological well-being of older individuals, especially of those living in areas of exceptional longevity such as the BZs.In addition, when the cognitive reserve has been evaluated, research often did not encompass participants’ whole lifespans. Therefore, this study intended to face these issues and its main goal was to explore the influence of cognitive reserve as a mediator in the relationship between the objectively assessed functional reserve (i.e., which can be defined as the difference between the maximum physical or mental capacity of a construct and the minimum necessary to perform daily functioning, [36]) and self-reported depressive symptoms of older individuals living in the Sardinian BZ. Moreover, this investigation also intended to examine the impact of cognitive reserve on the measures of functional reserve and self-reported mental health respectively, and the effect of gender on the cognitive and functional reserves of our participants. To achieve the second objective, a good vs. poor ability design was adopted, since it has been suggested that this approach is successful in examining individual differences [37]. Following previous research, the following hypotheses were yielded: (1) cognitive reserve was expected to be associated with functional health measures [32]; (2) the general Cognitive Reserve Index by Nucci et al. [29], and especially the measure of leisure time, were expected to be negatively associated with a self-report measure of depression [30]; (3) higher amount and intensity of PA were expected to be negatively associated with self-reported depressive symptoms [20, 33]; (4) engagement in leisure activities was expected to predict self-reported depressive symptoms [e.g. 24]; (5) specific indexes of cognitive reserve, such as years of education and occupation, were expected to be better in males than in females [38]; (6) the amount and intensity of performed PA were expected to be different in men and women [39–42]. Finally, due to the lack of previous findings, no further a priori predictions were provided on the mediational role of cognitive reserve between the functional reserve and self-reported depressive signs.

## Method

### Participants

One hundred and twenty 65-101-year-old participants, 52 males and 68 females (M_age_ = 82 years, SD = 8.4 years) were recruited in several villages of the Sardinian BZ. To take voluntarily part in the study, the following inclusion criteria had to be satisfied: (1) to be community-dwellers; (2) to have been born in the Sardinian BZ and be a permanent resident in that area; (3) do not exhibit any neurologic or musculoskeletal condition that might negatively impact walking; (4) to exhibit a Mini-Mental State Examination [43] score (see the Materials section) > 20 as lower values would indicate the presence of cognitive impairment. Gender was counterbalanced across the participants (χ² = 2.133, df = 1, p = .144). The mean Mini-Mental State Examination score in the current sample was 25.8 (SD = 2.07), that is, 113 participants were cognitively healthy and the remaining 7 exhibited some suspected signs of cognitive decline.

### Materials

The following battery of tests was presented:

The Mini-Mental State Examination (MMSE) [43] was used as a screening measure to evaluate global cognitive function through a set of items assessing distinct cognitive processes (e.g., spatiotemporal orientation, working memory, long-term memory). The maximum total score is 30 and a score included between 23 and 20 was used to identify participants with suspected signs of cognitive decline, whereas a score ≥ 24 indicates that the examinee is cognitively healthy. Following Magni et al. [44], the raw scores were adjusted for age and years of education.

The Cognitive Reserve Index questionnaire (CRIq) [29] was administered to evaluate three dimensions of cognitive reserve: educational attainment, level of work occupation (i.e., unskilled or manual work; skilled manual work; skilled nonmanual or technical work; professional occupation; highly intellectual occupation), and engagement in leisure (i.e., physical, social, and cultural) activities. This questionnaire is composed of 20 items organized into three sections. Apart from a total score (i.e., CRIq-tot), three further indexes relative to the years of education (i.e., CRIq-Edu), occupational level (i.e., CRIq-Job), and time spent for leisure (i.e., CRIq-Hobby) were computed. According to the original procedure, the raw scores were standardized using a scale with M = 100, and SD = 15. Thus, a score < 70 is very low, a score > 130 is very high, and a score included between 85 and 129 is considered medium. This questionnaire showed good internal consistency expressed by 0.73 Cronbach’s alpha coefficient [29].

The shorter version of the Geriatric Depression Scale (GDS) [45, 46] was designed to assess self-reported depressive signs. This scale encompasses 15 dichotomous items that do not describe somatic symptoms. A score > 10 indicates the occurrence of very significant depressive symptoms. In the current sample, the internal consistency of the scale is expressed by a split-half reliability = 0.74.

Finally, the functional reserve was assessed in terms of daily ambulation and performed PA (considering both amount and intensity), which were objectively assessed using a clinically validated three-axial accelerometer (Actigraph GT3X, Actigraph Co., Pensacola, FL, USA) previously employed in studies involving older adults [47–49].

### Procedure

Written informed consent was provided by each participant before the start of the testing session. Each person was individually tested in a quiet room of his/her home. After that the Mini-Mental State Examination was administered, some socio-demographic information (e.g., marital status, gender) was collected and then the CRI-q and GDS were proposed. This experimental session lasted approximately 40 min.

At the end of the psychological session, participants were required to wear the accelerometer on their non-dominant wrist for 7 consecutive days and were instructed not to remove it except in case of showering, bathing, and when performing water-based activities (i.e., swimming). The choice of the wrist as the placement site was made to reduce discomfort, increase the likelihood of wear time compliance and make data collection on sleep patterns easier [50, 51]. Accelerometric data were collected in 60-s epochs at 30 Hz frequency. At the end of the acquisition period, the raw data were downloaded on a PC and processed using the dedicated ActiLife software (v6.13.3 Actigraph Co., Pensacola, FL, USA) to extract the following variables:

- wear time (i.e., the number of hours which refers to valid accelerometric data).
- step counts (average weekly value).
- PA intensity, calculated as the percentage of time spent in each of the following three categories defined according to the associated value of metabolic equivalent (MET): sedentary behavior (SB, 0–1.5 MET), light intensity PA (LPA, 1.5–3 MET) and moderate-to-vigorous PA (MVPA, > 3 MET). Such discrimination was carried out using the cut points for accelerometric counts per minute (cpm) proposed by Migueles et al. [52]. In the analysis, we considered and included only acquisition days characterized by a wear time of at least 16 h.

### Statistical Analyses

Statistical analyses were carried out using version 2.3 of the Jamovi open-source package [53] and SPSS 24.0. The 0.05 statistical significance was set throughout all the analyses. Descriptive statistics were performed to explore the sociodemographic characteristics of the participants and their performances in the psychological and motor measures. Pearson’s coefficients were calculated to investigate the nature of the associations between self-reported depression, cognitive reserve, and functional reserve indexes. Then, based on the outcomes of these analyses, three mediation analyses were performed to explore whether the relationship between self-reported depressive symptoms and the functional reserve was mediated by the cognitive reserve. Specifically, in each analysis, the dependent variable was the GDS score, number of daily steps, and time spent in each level of PA intensity (i.e., functional reserve) were respectively used as predictors, whereas the impact of the cognitive reserve as a mediator was examined using both the CRIq-tot and CRIq-Hobby scores, respectively. The significance of the direct and indirect effects was tested. In each condition, the indirect effect was tested using a bootstrap estimation approach with 5000 samples [54]. It was assumed that there was an effect of the cognitive reserve over the GDS score when the indirect effect was statistically significant (p < .05). Finally, to examine the impact of gender on cognitive reserve and functional reserve measures, a Multivariate Analyses of the Covariance (MANCOVA) and two distinct analyses of the covariance (ANCOVAs) were conducted, whilst controlling for the effect of global cognitive functioning.

## Results

Table 1 summarizes the correlational analyses conducted between GDS, cognitive reserve, and functional reserve measures, respectively.

**Table 1:** Pearson’s correlations among self-reported depressive symptoms (i.e., GDS), global cognitive reserve (i.e., CRIq-tot), cognitive reserve evaluated in terms of educational attainment (i.e., CRIq-Edu), occupation (i.e., CRIq-Job), time spent for leisure activities (i.e., CRIq-Hobby), and functional reserve assessed in terms of the number of daily steps (i.e., Steps) and percentage of time spent in physical activity of moderate-to-vigorous intensity (i.e., MVPA).

		GDS		CRIq-tot		CRIq-Edu		CRIq-Job		CRIq-Hobby		Steps		MVPA	
GDS		—													
CRIq-tot		-0.334	***	—											
CRIq-Edu		-0.162		0.696	***	—									
CRIq-Job		-0.235	*	0.826	***	0.527	***	—							
CRIq-Hobby		-0.326	***	0.730	***	0.256	**	0.299	**	—					
Steps		-0.252	**	0.209	*	0.170		0.066		0.258	**	—			
MVPA		-0.253	**	0.152		0.144		0.001		0.222	*	0.843	***	—	

Notes. * p < 0.05, ** p < 0.01, *** p < 0.001

**Fig. 1:**
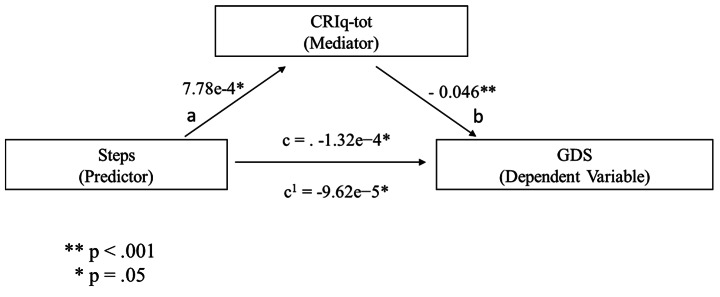
predicted mediational patterns for GDS. The CRIq-tot score was the cognitive reserve index used as a mediator, whereas the daily steps index (i.e., Steps) was used as a predictor

Based on the outcomes of the correlational analyses, three distinct mediation analyses were performed to establish whether the relationship between the functional reserve and self-reported depressive score was mediated by the cognitive reserve. As explained earlier, this is the main goal of the study. Thus, it was found that the number of daily steps (Steps) was a significant predictor of the CRIq-tot score [b = 7.78e-4, SE = 3.44e-4, p = .024 (path a in Fig. 1)], and CRIq-tot score significantly predicted the GDS index [b = − .0461, SE = .0134, p < .001 (path b in Fig. 1). The mediation hypothesis for the self-reported depressive score is supported. After controlling for CRIq-tot (path c’ in Fig. 1), Steps remained a significant predictor of the GDS score (b = -9.62e − 5, SE = 4.65e-5, p = .038). Furthermore, the indirect coefficient was significant [b = -3.59e − 5, SE = 1.61e-5, 95% CI = -6.85e − 5, -6.84e − 6; p = .026]. The standardized effect size indicated that 27.2% of the variance in the GDS condition was explained by Steps and CRIq-tot measures. Figure 1 illustrates these findings.

**Fig. 2:**
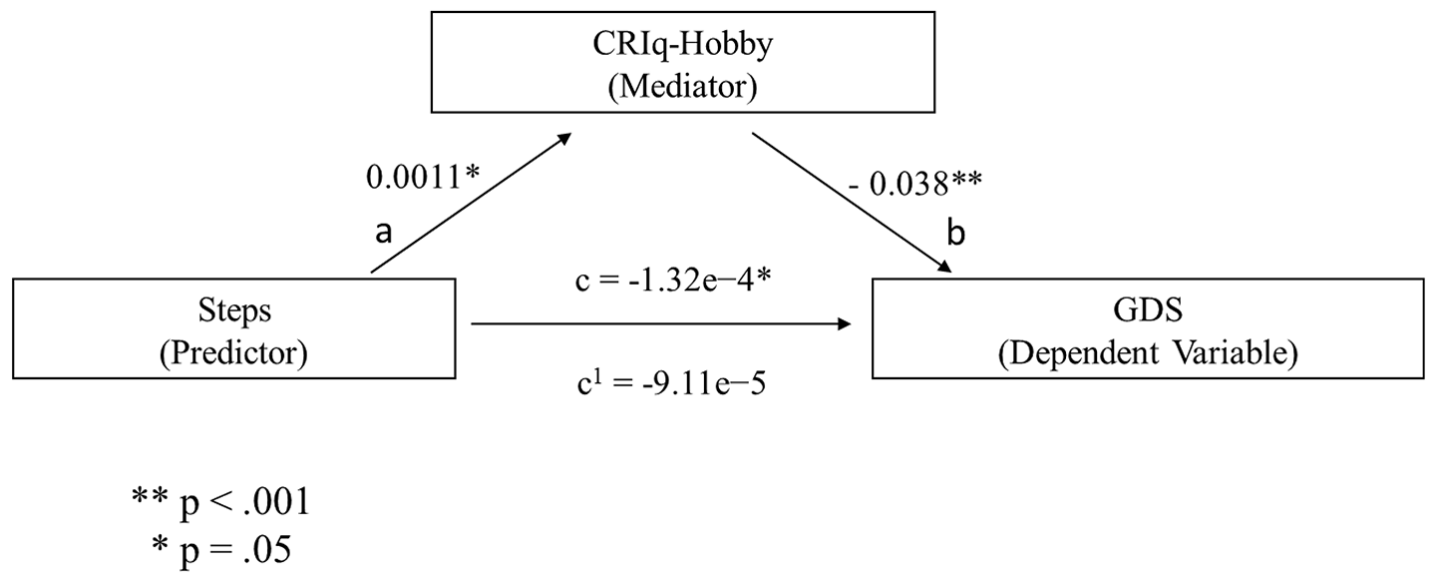
predicted mediational patterns for GDS. The CRIq-Hobby score was the cognitive reserve index used as a mediator, whereas the daily steps index (i.e., Steps) was used as a predictor

A similar mediational analysis was conducted, in which CRIq-Hobby was used as the mediator. The Steps measure significantly predicted CRIq-Hobby [b = .0011, SE = 3.69e-4, p = .003 (path a in Fig. 2)], as well as CRIq-Hobby, was a significant predictor of the GDS total score [b = − .038, SE = .0104, p < .001, (path b in Fig. 2)]. Therefore, the mediation hypothesis is supported. After controlling for the CRIq-Hobby effect (path c’ in Fig. 2), the Steps measure did not remain a significant predictor of GDS (b = -9.11e − 5, SE = 4.76e-5, p = .055). However, the indirect coefficient was significant: b = − 4.10e − 5, SE = 1.76e-5, 95% CI = -7.98e − 5, -1.16e − 5; p = .02]. The standardized effect size indicated that 31% of the variance in the GDS condition was explained by Steps and CRIq-Hobby. Figure 2 shows these outcomes.

**Fig. 3:**
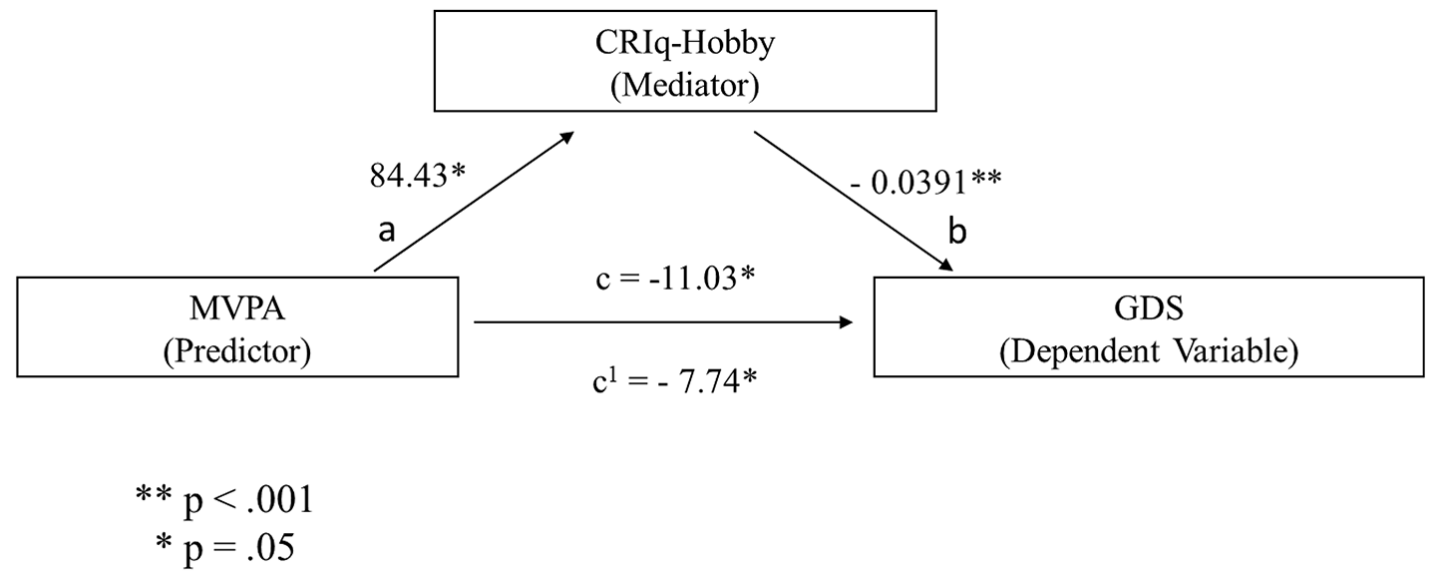
predicted mediational patterns for GDS. The CRIq-Hobby score was the cognitive reserve index used as a mediator, whereas the MVPA index was used as a predictor

The third mediation model showed that the percentage of time spent in PA of moderate-to-vigorous intensity (MVPA) significantly predicted the CRIq-Hobby score [b = 84.43, SE = 33.75, p = .012 (path a in Fig. 3)], and this cognitive index predicted the GDS score [b = − .0391, SE = .0104, p < .001 (path b in Fig. 3)], confirming that the mediation hypothesis is supported. After controlling for the CRIq-Hobby impact (path c’ in Fig. 3), the MVPA index remained a significant predictor of the GDS score (b = -7.74, SE = 3.73, p = .038). Moreover, the indirect coefficient was significant [b = -3.30, SE = 1.54, 95% CI = -6.64, − 0.676; p = .032]. The standardized effect size indicated that 30% of the variance in the GDS condition was explained by MVPA and CRIq-Hobby. These results are illustrated in Fig. 3.

Then, to pursue the second goal, a subgroup of participants (n = 7) exhibiting low CRIq-tot score (i.e., a score < 76) and another subsample of participants (n = 10) with high CRIq-tot resources (i.e., a score > 125) were identified. These two subsamples were similar in terms of numerosity (χ² = 0.529, df = 1, p = .467), age [U = 17.5, z = − 1.713, p = .087], and MMSE scores [U = 17, z = − 1.764, p = .078]. Moreover, three Mann-Whitney U tests revealed that participants with low global cognitive reserve reported more depressive signs (Md = 12.5), made fewer Steps (Md = 4.57), and exhibited less MVPA (Md = 5.14) than participants reporting higher CRIq-tot scores (Md for GDS = 6.55, U = 10.5, z = − 2.453, p = .014; Md for Steps = 12.10, U = 4, z = − 3.025, p = .002; Md for MVPA = 11.7, U = 8, z = − 2.635, p = .007).

In addition, to pursue the third objective, a between-subjects MANCOVA was performed to investigate the impact of gender on the CRIq indexes, using the MMSE score as the covariate. The Multivariate tests revealed the significant main effects of MMSE [Wilks’ λ = 0.91, df = 4;107, p = .03] and gender [Wilks’ λ = 0.26, df = 4;107, p < .001]. The main effect of gender was found in the CRIq-tot [F(1,110) = 11.654, p = .001, η²_p_ = .10], CRIq-Job [F(1,110) = 35.751, p < .001, η²_p_ = .24], and CRIq-Hobby [F(1,110) = 6.08, p = .015, η²_p_ = .05] conditions but not in the CRIq-Edu one [F(1,110) = 0.504, p = .48]. Overall men reported better cognitive reserve than women. Finally, two ANCOVAs were performed to examine the gender effect on the functional reserve measures, whilst controlling for the effect of MMSE. In the MVPA condition, there were the main effects of both gender [F(1,106) = 7.341, p = .008, η²_p_ = .06] and MMSE [F(1,106) = 4.93, p = .029, η²_p_ = .04]. Males exhibited lower MVPA (M = 0.041, SD = 0.03) than females (M = 0.063, SD = 0.05). In contrast, in the Steps condition, there was the main effect of MMSE [F(1,106) = 7.023, p = .009, η²_p_ = .06], where the effect of gender was not significant [F(1,106) = 2.562, p = .112]. Table 2 summarizes the mean scores of male and female participants in each measure of cognitive and functional reserves.

**Table 2:** Mean scores of the male participants and females in global cognitive reserve (i.e., CRIq-tot), educational attainment (i.e., CRIq-Edu), occupation (i.e., CRIq-Job), time spent for leisure activities (i.e., CRIq-Hobby), Steps, and MVPA conditions. M denotes the mean score, whereas SD refers to the standard deviation

	Males	Females	Males vs. Females p	η²p
CRIq-tot	M = 98.65 (SD = 17.13)	M = 89.15 (SD. = 12.94)	0.001	0.10
**CRIq-Edu**	M = 97.69 (SD = 11.23)	M = 94.65 (SD = 9.4)	0.48	
CRIq-Job	M = 96.65 (SD = 19.84)	M = 84 (SD = 15.5)	< 0.001	0.24
CRIq-Hobby	M = 102.5 (SD = 18.33)	M = 96.8 (SD = 16.73)	0.015	0.05
Steps	M = 87.94 (SD = 4212.71)	M = 100.66 (SD = 4246.26)	0.112	
MVPA	M = 0.04 (SD = 0.035)	M = 0.063 (SD = 0.052)	0.008	0.06

## Discussion

The main objective of the present investigation was to provide insights into the role played by cognitive reserve as a mediator between self-reported depression and functional reserve in people living in an area of successful aging and exceptional longevity, the Sardinian BZ. Besides, this study also intended to examine the impact of gender on some measures of cognitive and functional reserves in late adulthood, while the effect of global cognitive functioning was controlled for. To our knowledge, the novelty consists in the fact that this is the first study in which the cognitive reserve of older participants living in a BZ was assessed by a validated questionnaire that examines one’s whole lifespan and so far, this is the first study that concurrently investigated the interplay between cognitive reserve, objectively assessed functional reserve and self-reported mental well-being in a sample of older people recruited in an area of exceptional longevity such as the Sardinian BZ. From an applied perspective, these goals are crucial since they may contribute to shedding light on the implementation of actions promoting HRQoL and therefore successful aging.

Following Cohen [55], and consistent with Coin et al. [30], statistically significant small and medium negative correlations have been found between cognitive reserve (i.e., the general cognitive reserve index and those relative to the job occupation and the engagement in leisure activities) and self-reported depressive signs, suggesting that older people exhibiting better cognitive reserve is less exposed to the risk to develop depression. Moreover, as expected [20, 33], negative associations have been found between two measures of motor efficiency (i.e., Steps and MVPA) and self-reported depressive signs. In addition, following Quattropani et al. [32], it has been documented that cognitive reserve was positively associated with functional health. Overall, these results suggest that people with decreased mood tend to be less engaged with socio-cultural life, are less efficient from a physical viewpoint, and exhibit fewer cognitive resources to compensate for the impact of age-related factors on their physical and mental functioning. However, extending previous findings concerning the impact of some dimensions of cognitive reserve on the subjective psychological well-being of older people living in areas of exceptional longevity [e.g., 20, 22, 24, 25], the most innovative result of this investigation is that cognitive reserve assessed on one’s lifespan mediates between motor efficiency and self-reported depression of older people of the Sardinian BZ. Specifically, it has been documented that a significant proportion of the variance in the GDS condition (i.e., between 27.2% and 31%) was explained by some measures of mobility (i.e., Steps and MVPA) and cognitive reserve (i.e., both the general index and that relative to the engagement in leisure activities). In line with these outcomes, it has also been shown that people who reported the lowest global cognitive reserve also exhibited worse functional health and higher self-reported depressive symptoms. Therefore, embracing an applied perspective, these outcomes seem to corroborate the view of the cognitive reserve as a key factor in sustaining some dimensions of HRQoL (i.e., physical health and psychological well-being) in old age [56]. Moreover, partially following previous findings [38], it has been documented that men reported better global cognitive reserve and better job occupation, as well as they spent more time in outdoor leisure activities than females. These outcomes can be justified by the fact that in the Sardinian BZ, older men are traditionally in charge to provide economic support to their families (i.e., they were either shepherds or peasants) [57]. However, despite our hypothesis, men were not more educated than women, perhaps because in the rural area where our participants were recruited, historically males were engaged since early childhood in the cultivation of the land or as servants in taking care of livestock, whereas women were mainly devoted to the household [58]. This could explain why our male and female participants exhibited modest levels of education. Overall, it seems plausible to conclude that in a rural context such as that of the Sardinian Blue Zone, the contribution of educational attainment in the enhancement of the cognitive reserve of older individuals is quite modest, since CRIq-Edu was not significantly associated with the self-reported depressive score and the objectively assessed measures of functional reserve. Despite this, it is noteworthy that compared to the standardized values proposed by Nucci et al. [29], both our male and female participants exhibited preserved cognitive reserve (i.e., mean scores).

As regards the PA parameters, our data revealed that compared to men, women were more active in terms of both daily taken steps and the percentage of time spent in MVPA. The effect of gender on the amount and intensity of performed PA is still not completely clear, as mixed findings are reported in the literature. For instance, the Seniors-ENRICA-2 study recently performed in Spain using the same accelerometric setup of the present study, reported that compared to men, women were characterized by significantly lower levels of SB and higher LPA, although no differences were observed in terms of MVPA [41], as already reported in previous studies carried out in Iceland, Norway, and Japan [39, 59, 60]. However, the results of the large study carried out by Chen et al. [40] on 1739 individuals revealed that compared with men, women accumulated more minutes of LPA and MVPA and spent less time in SB, while mean steps per day did not differ significantly. A similar trend was also observed in the Rotterdam study by Koolhaas et al. [61] who tested 1210 individuals aged 70–94 and detected that men spent significantly more time in SB and less time in LPA and MVPA than women. At last, it should be noted that the meta-analysis by Webster et al. [42] concluded that, among individuals aged over 80, men spend a significantly larger proportion of time in SB. While reasons for such discrepancies can be partly due to different equipment used in the studies, site of positioning, and cut-points adopted to classify the intensity of PA, several authors have hypothesized that factors like cultural lifestyle differences and the presence of chronic diseases able to impact PA (men are more prone to be affected by cardiovascular diseases, stroke, and diabetes) [62] might play a relevant role in determining the above-mentioned gender differences. In this context, our results seem to be consistent with those observed in Japan, probably because the rural environment and socio-economic features of the small villages of the Sardinian BZ where our participants were recruited, are associated with a traditional lifestyle that is different from those typical of most Western countries. A further reason could be found in the cultural heritage and traditional gender roles (i.e., women are more involved in household activities than men) that persist in those Sardinian rural communities [58].

However, caution is needed, given the exploratory nature of this study. Indeed, the current investigation does have several limitations, including the sample size, the limited battery of tasks evaluating the mental well-being and functional status, as well as the fact that older individuals were cognitively healthy community dwellers that were recruited only in the Sardinian Blue Zone limits. Finally, it must also be considered that this is not a longitudinal study, therefore we cannot conclude what the developmental trends of mental well-being and functional status of our participants were. These aspects limit the generalizability of the current outcomes, therefore current findings cannot be extended to people living in other BZ, those institutionalized, and to older individuals living in areas not characterized by the exceptional longevity of their inhabitants. Therefore, future research should face these issues, replicating the study with wider samples of older typically and atypically developing participants (e.g., cognitively healthy individuals and those exhibiting Mild Cognitive Decline) living in their own homes or nursing homes. Moreover, future studies should use a wider battery of tasks, including those evaluating hedonic and eudaimonic well-being and further motor tasks examining other functional parameters such as gait speed and muscular strength.

## Conclusions

In conclusion, the findings of this study extend those of previous investigations conducted in the BZs [e.g., 14, 15, 35] and suggest that the implementation of specific interventions aimed at reinforcing cognitive reserve along the whole lifespan is crucial for the enhancement of HRQoL in very late adulthood. In this regard, as reported in the literature [e.g., 63], the combination of moderate physical exercise with socially oriented and cognitively stimulating recreational activities (e.g., courses to learn a foreign language, reading and commenting on a book with a group of peers, learning to play a music instrument) regularly performed is very useful for the maintenance of an active and healthy lifestyle. This would be very beneficial, especially for older individuals who are very sedentary (i.e., no time spent per week performing physical exercise or other leisure activities) and therefore exhibit less preserved cognitive efficiency (e.g., poorer executive functions, immediate recall from working memory) [64, 65], and those who in their professional life were mainly engaged in the very high-physically demanding jobs [63]. As pointed out by Li et al. [65], nowadays 50% of older people in the USA are physically inactive and at risk of developing cognitive deterioration. Therefore, participation in late-life leisure activities promoting social functioning, and physical and cognitive reserves is essential to prevent and delay, when possible, the cognitive decline, to compensate for the physiological cognitive loss (e.g., learning new coping strategies to apply in daily life), and boost the independency (i.e., by the reinforcement of motor functioning and cognitive reserve through the proposed cognitively stimulating and physically demanding activities) of older people. Finally, the multidomain interventions aimed at empowering functional and cognitive reserve in the later lifespan are essential to limit the hospitalizations of more sedentary older individuals and therefore to contain the public expenses allocated for the promotion of health in the last decades of life [66].

### Electronic Supplementary Material

Below is the link to the electronic supplementary material.

Supplementary Material 1

## Data Availability

The data that support the findings of this study are not publicly available due to privacy or ethical restrictions.
