# Supplementary material for: Is Cognitive Reserve a Determinant of Functional and Mental Health in Older People of the Sardinian Blue Zone? A Mediational Approach
Source: Psychiatr Q. 2023 Aug 29;94(4):617–32. doi: 10.1007/s11126-023-10047-6 (PMC10638121; doi:10.1007/s11126-023-10047-6)
Supplement: Supplementary file 1 — Supplementary Material 1 [file 11126_2023_10047_MOESM1_ESM.pdf]

Springer
the language of science

Disclosure of Interest Form

Psychiatric Quarterly

When an author or the institution of the author has a relationship, financial or otherwise, with individuals or organizations that could influence the author's work inappropriately, a conflict of interest may exist. Examples of potential conflicts of interest may include but are not limited to academic, personal, or political relationships; employment; consultancies or honoraria; and finical connections such as stock ownership and funding. Although an author may not feel that there are conflicts, disclosure of relationships and interests that could be viewed by others as conflicts of interest affords a more transparent and prudent process.

All authors for *Psychiatric Quarterly* must complete this form and disclose any actual or potential conflict of interest. The Journal may publish such disclosures if judged to be important to readers.

Please complete and return this form to Editor Jeffrey Borenstein at psychiatricquarterly@gmail.com.

☑ I have no potential conflict of interest pertaining to this *Psychiatric Quarterly* submission.

Category for Disclosure	Description of Interest/Arrangement

Author Name Maria Chiara Fastame

Article Title Is Cognitive Reserve a Determinant of Functional and Mental Health in Older People of the Sardinian Blue Zone? A mediational approach

Author Signature [signature] Date 20/05/2023

http://www.springer.com/journal/11126

Psychiatric Quarterly
Editor: Borenstein, J.
ISSN: 0033-2720 (print version)
ISSN: 1573-6709 (electronic version)
Journal no. 11126
